# Sexual harassment exposure among junior high school students in Norway: prevalence and associated factors

**DOI:** 10.3389/fpubh.2024.1307605

**Published:** 2024-01-18

**Authors:** Tore Bonsaksen, Anne Mari Steigen, Marie Dahlen Granrud, Cecilie Ruud Dangmann, Tonje Holte Stea

**Affiliations:** ^1^Department of Health and Nursing Science, Faculty of Social and Health Science, Inland Norway University of Applied Sciences, Elverum, Norway; ^2^Department of Health, Faculty of Health Science, VID Specialized University, Stavanger, Norway; ^3^Department of Social Sciences and Guidance, Faculty of Social and Health Science, Inland Norway University of Applied Sciences, Elverum, Norway; ^4^Department of Health and Nursing Sciences, University of Agder, Kristiansand, Norway

**Keywords:** adolescents, depressive symptoms, loneliness, Norway, prevalence, self-esteem, sexual abuse, sexual harassment

## Abstract

**Background:**

Sexual harassment is common in most countries and cultures. Less is known about sexual harassment, its risk factors, and psychosocial outcomes among young adolescents. The aim of the study was to examine the 12-month prevalence of sexual harassment exposure, and sociodemographic and psychosocial factors associated with exposure among junior high school students in Norway.

**Methods:**

A comprehensive cross-sectional study was completed by 83,297 Norwegian adolescents in junior high school in 2021. Data on exposure to sexual harassment were used in combination with sociodemographic measures and psychosocial outcomes: depressive symptoms, loneliness, self-esteem, and well-being. Group differences were analyzed with Chi Square tests, and sociodemographic risk factors were analyzed with logistic regression. Psychosocial outcomes were assessed with multivariate and univariate ANOVAs, and dose–response relationships were assessed with linear regression.

**Results:**

Among the adolescents, 32.6% had experienced sexual harassment during the past year. Girls had higher odds of exposure (OR: 1.90, 95% CI: 1.84–1.96), whereas adolescents whose parents had higher education had lower odds (OR: 0.95, 95% CI: 0.90–0.99). Exposure was associated with higher levels of depressive symptoms (partial η^2^ [ES] = 0.11) and loneliness (ES = 0.07), and with lower self-esteem (ES = 0.06) and well-being (ES = 0.06). Among exposed adolescents, more frequent exposure was associated with poorer outcomes.

**Conclusion:**

Exposure to sexual harassment affects one third of junior high school students in Norway. Those who are exposed have poorer psychosocial outcomes, and there is a dose–response relationship between exposure and outcomes. Awareness of sexual harassment among young adolescents, and interventions to prevent and reduce it, are needed.

## Introduction

Sexual harassment is rooted in historic power imbalances, and the #MeToo movement increased public awareness of how it is pervasive across cultures, age groups and genders (1). It is commonly classified into three distinct forms (2, 3). These are gender harassment (insulting remarks or behavior, intending to provoke negative emotions), unwanted sexual attention (uninvited and explicit display of sexual desire or intention towards the other person), and sexual coercion (applying any form of pressure to elicit sexual cooperation). Thus, sexual harassment can encompass a broad range of behaviors which include physical contact with the victim as well as interactions based on verbal exchange (4). Common verbal forms of sexual harassment include sexually laden name-calling (such as “whore” or “poof”) and making unwanted sexual comments or jokes, but non-contact sexual harassment also includes peeping and spreading sexual rumors (5). In recent years, the online communication platforms have created new challenges, such as sexting (sending sexual text messages often including nude or seminude photos or videos) and online sexual harassment (6). Pictures and videos portraying nudity can easily be disseminated online (7, 8), to the extent that adolescents may be reluctant to shower after gym classes in fear of being exposed to this kind of harassment.

While gender equality and non-discrimination have been on the political agenda in Norway for decades, exposure to sexual harassment is commonplace. Based on previous studies, a recent rapid review of sexual harassment in Norway reported an overall lifetime prevalence of 8% (9). However, large variations were reported between different professional groups (engineers 1%, nurses 20%), women were more often exposed than men, and younger people more often than older (9). Among adolescents, international studies have reported higher, but widely varying, prevalence rates of sexual harassment exposure [e.g., (10–14)]. They have been consistent, however, in reporting that girls are at higher risk of exposure than boys. Among adolescents in Norway, similar rates of exposure to non-contact forms of sexual harassment during the past 12 months were found among boys (62%) and girls (63%) in 2014 (5), whereas substantially lower rates, in particular among boys (boys 26.5% versus girls 36.1%), were found in another Norwegian study a few years later (15). As the two Norwegian studies (5, 15) were conducted a few years apart, and in different parts of the country, both time trends and differences in sample composition are possible explanations for the different results reported.

In addition to female gender, a number of risk factors for sexual harassment exposure among adolescents have been reported. For example, risk factors have included racial (16) and sexual minority identity (17), emotional problems (18), and higher frequency of alcohol use and delinquent behaviors (17, 19). Higher risk of sexual abuse (20) and online sexual abuse (21) has been found among adolescents in less affluent families, and lower parental education levels have been associated with higher likelihood of adolescents reporting sexual harassment experience (22).

The negative outcomes related to (traditional or online) sexual harassment exposure among adolescents may be both short-term and long-term and may pose a direct threat to the individual’s health and well-being. Studies have reported various negative psychosocial outcomes, including symptoms of anxiety and depression (6, 14, 23, 24), and that concurrent alcohol and drug use can make the symptoms of mental health problems worse (24). Victims of bullying in general, including sexual bullying, may tend to withdraw from social encounters with peers and feel like an outsider when involved in social situations (25), which in turn may give rise to loneliness. As social anxiety has been temporally linked to subsequent loneliness in adolescents (26), sexual harassment may also be indirectly related to loneliness through social anxiety. People who have been exposed to sexual harassment as adolescents have also been shown to be more likely to have a higher number of sex partners and to contract a sexually transmitted disease in early adulthood (27), and may be more likely to develop obesity (11, 28). Outcomes are likely to vary with the severity and frequency of harassment experiences as well as the psychological resilience among those exposed (29). Thus, adolescents may be particularly vulnerable to experiencing a range of psychosocial problems following sexual harassment. Continued research efforts are needed to gain a more comprehensive understanding of risk factors and psychosocial consequences of sexual harassment in this vulnerable population.

While there is a vast number of studies on sexual harassment and its consequences, most studies appear to focus on older adolescents and young adults, such as students in higher education (30). Less is known about the prevalence of sexual harassment, its risk factors, and psychosocial outcomes among young adolescents in junior high school. However, in line with the view purported by Collinsworth and co-workers (29), one might expect this youngest group of adolescents to be the least resilient to adverse events, and thus experience worse outcomes of sexual harassment when exposed. The youngest adolescents may also be less able to identify their experience as one of sexual harassment victimization, as a previous study showed that the ability to identify and verbalize such experiences increases with age and may not be fully developed before well into adulthood (31). The ability to identify and verbalize sexual harassment experiences is also vital for victims’ ability to seek and make use of appropriate support from adults. Moreover, few studies have concurrently examined a variety of psychosocial outcomes and their associations with sexual harassment exposure. Thus, to gain knowledge about precursors and consequences of sexual harassment, a wider perspective is needed. The aim of this study is to examine the 12-month prevalence of sexual harassment exposure, and sociodemographic (age, gender, parents’ education level, centrality of place of living, family affluence) and psychosocial factors (depressive symptoms, loneliness, self-esteem, well-being) associated with exposure to sexual harassment among junior high school students in Norway. Further, among those exposed to sexual harassment, we examine possible linear patterns in the relationships between frequency of harassment and psychosocial factors.

## Methods

### Survey and procedure

A comprehensive cross-sectional study, the Ungdata survey, was in 2021 completed by 139,841 Norwegian adolescents aged between 13 and 19 years. The survey is conducted annually across most Norwegian municipalities and is an essential source of information on young peoples’ health, well-being, attitudes, and behaviors across a range of areas (see www.ungdata.no). [Removed for peer review] is responsible for the survey in a collaboration with the Regional Drug and Alcohol Competence Centers (KoRus). The surveys are financed partially by the Norwegian Directorate of Health.

Parents and adolescents were informed via mail prior to the data collection, and the parents were assured that they could withdraw their children from participation at any time. The adolescents decided in school whether they wanted to participate after being informed that participation was voluntary and that they could skip questions that they did not want to answer. The study was conducted as a web-based questionnaire administered at school during school hours with a teacher or an administrator present to answer questions. The adolescents used approximately 30–45 min to complete the questionnaire.

### Measures

#### Sexual harassment

Sexual harassment was assessed with the following question: “Over the past 12 months, have you been subjected to any of the following in a manner that you absolutely did not like?” Then, three forms of harassment were listed: (i) “That someone has touched me in a sexual way against my will,” (ii) “That someone in a hurtful manner has called you a whore, poof, or other words with a sexual content”; and (iii) “That someone has spread negative sexual rumors about you.” On each item, response options never (1), once (2), 2–5 times (3), and 6 or more times (4). These items have been used consistently in the Ungdata surveys since 2017 to assess sexual harassment exposure among adolescents (32).

Each of the items were later dichotomized to distinguish between adolescents who had not been exposed to the sexual harassment form (response option 1) and those who had been exposed (response option 2–4). Further, a variable labelled “any sexual harassment”, distinguishing between those never exposed to any kind of sexual harassment (0) and those exposed to one of more kinds at one or several occasions (1), was computed.

#### Depressive symptoms

Depressive symptoms were measured using a six-item scale derived from the Depressive Mood Inventory (33), in turn based on the Hopkins Symptom Checklist (34). The adolescents were asked if they had been affected by any of the following during the past week: “Felt that everything is a struggle” (item 1), “had sleep problems” (item 2), “felt unhappy, sad or depressed” (item 3), “felt hopelessness about the future” (item 4), “felt stiff or tense” (item 5), “worried too much about things” (item 6). Four response categories were applied to each of the six items: “Not been affected at all” (1), “not been affected much” (2), “been affected quite a lot” (3), and “been affected a great deal” (4). Sum scores were computed, ranging from 6 to 24, where higher scores indicated higher levels of depressive symptoms. The scale has been psychometrically evaluated among Norwegian adolescents in previous Ungdata-based studies, demonstrating good reliability (Person Separation Index: 0.802) and appearing overall to work reasonably well (35).

#### Loneliness

Loneliness was measured with one item. It had an opening phrase identical to the items included in the depressive symptoms scale (i.e., “During the past week, have you been affected by any of the following issues”), and then stated, “felt lonely.” Response options were “not been affected at all” (1), “not been affected much” (2), “been affected quite a lot” (3), and “been affected a great deal” (4) (32). Thus, scores ranged between 1 and 4, with higher scores signifying higher levels of loneliness.

#### Self-esteem

Self-esteem was assessed with one item: “I like myself the way I am,” with response options “not at all true” (1), “not very true” (2), “quite true” (3), and “very true” (4), so that higher scores indicate higher self-esteem. The item is derived from the “global self-worth” subscale of the Self-Perception Profile for Adolescents (36).

#### Well-being

A modification of Cantril’s ladder, as adopted in the Gallup World Poll (37), was used as a measure of subjective well-being. The adolescents were asked to rate their level of present-time well-being on a scale from 0 to 10, where 0 indicates worst possible well-being and 10 means best possible well-being.

#### Sociodemographic variables

Sociodemographic variables included in this study were gender, reported as male or female. While age is not assessed directly in the Ungdata surveys, grade was used as an indicator for age. Parental education was measured by asking the pupils whether none, one or both parents had higher education. In the analysis, this variable was dichotomized into “none of the parents have higher education” and “at least one parent has higher education”. Centrality, as defined by Statistics Norway (38), was used as a proxy for how centrally located a municipality in Norway is. The measure is based on how many jobs and service institutions can be reached by car within 90 min from where one lives. All municipalities are categorized on a scale from 1 (most central) to 6 (least central).

The Family Affluence Scale (39, 40), version II, was used to assess the socioeconomic status of the adolescents. The measure is comprised of four questions: (i) Does your family have a car? where response options are “no”, “yes one”, and “yes, two or more”; (ii) Do you have your own bedroom? with response options “yes” or “no”; (iii) How many times have you travelled somewhere on holiday with your family over the past year? with response options “never”, “once”, “twice”, and “more than twice”; and (iv) How many computers or tablet computers does your family have?, where response options were “none”, “one”, “two”, and “more than two”. The addition “…or tablet computers” in question (iv) was added by the Ungdata administration in 2017 (32). All items were coded so that higher scores indicated higher levels of affluence. A sum score based on all four items was calculated and used as an overall measure of family affluence, with scores ranging between 4 (lowest affluence) and 13 (highest affluence). Validation studies have indicated high levels of child–parent agreement on scale items and on the whole scale, except for the item regarding holiday (39). Another study, using data from 25 countries, found the aggregated FAS at the country level to correspond well with the national wealth indicator, indicating good criterion validity (41).

### Data analysis

Participants with missing data were removed from the analyses casewise (analysis by analysis), with the exception of the multivariate analyses which required no missing data on the employed variables. Thus, sample size varied between analyses.

Descriptive analyses were included for all variables. Cross-tabulation of independent variables with sexual harassment was analyzed with Chi-square tests. Based on the initial cross-tabulation, a logistic regression analysis was performed to examine multivariate associations with sexual harassment exposure. To examine possible psychosocial consequences of sexual harassment exposure, a multivariate analysis of variance (MANOVA) was performed using depressive symptoms, self-esteem, loneliness, and subjective well-being as dependent variables, while using sexual harassment, gender, and the interaction term gender × sexual harassment as predictors. In the case of a statistically significant MANOVA, consecutive univariate ANOVAs followed for each dependent variable using the same set of predictors.

Among those exposed to sexual harassment, possible linear patterns in the relationships between frequency of harassment and psychosocial factors were examined with linear regression analyses. A “harassment type × gender” interaction term was included to examine the possibility of relationships varying between boys and girls. For loneliness and self-esteem, which were measured with one-item scales with relatively few response options (scale range 1–4), analyses of dose–response relationships were also conducted with ordinal regression analyses. For all analyses, statistical significance was *p* < 0.05. Effect sizes were interpreted as suggested by Pallant (42) and Cohen (43): partial η^2^ about 0.01 and standardized β about 0.10 indicate a small effect, partial η^2^ about 0.06 and standardized β about 0.30 indicate a medium effect, and partial η^2^ about 0.14 and standardized β about 0.50 indicate a large effect. In the ordinal regression analyses, estimates of associations (compared with the reference category) were reported along with the corresponding 95% confidence intervals (CI).

### Ethics

The study was conducted in accordance with the Declaration of Helsinki. Before participating in the study, all adolescents were asked to provide informed consent. The parents received oral and written information about the study and were given the opportunity to withdraw their children from participation. The information letter was approved by The Norwegian Centre for Research Data (NSD). All data were collected anonymously and then analyzed by independent researchers who did not participate in the collection of the data. The study was approved by the Research Ethics Committee at Inland Norway University of Applied Sciences (protocol code 21/01894).

## Results

### Participants

The study extracted a sample of adolescents (*n* = 83,297) in junior high school, aged 13–16 years. The response rate was 83% (44), and the gender proportions were similar. About one third of the adolescents were enrolled in each of the three grade cohorts, and the vast majority had parents among whom at least one had higher education. The description of the sample is shown in Table 1.

**Table 1:** Sexual harassment exposure in sample subgroups.

Characteristics	Total *n*	Exposed *n* (%)	Not exposed *n* (%)	*p*
Grade				
8th grade	25,655	8,205 (32.0)	17,450 (68.0)	0.06
9th grade	25,369	8,295 (32.7)	17,074 (67.3)	
10th grade	24,949	8,212 (32.9)	16,737 (67.1)	
Missing	7,324 (8.8%)			
Gender				
Boys	38,642	9,724 (25.2)	28,918 (74.8)	<0.001
Girls	38,403	15,014 (39.1)	23,389 (60.9)	
Missing	6,252 (7.5%)			
Centrality				
1 Most central	10,565	3,212 (30.4)	7,353 (69.6)	<0.001
2	18,013	5,918 (32.9)	12,095 (67.1)	
3	19,744	6,580 (33.3)	13,164 (66.7)	
4	16,207	5,241 (32.3)	10,966 (67.7)	
5	10,291	3,345 (32.5)	6,946 (67.5)	
6 Least central	4,125	1,417 (34.4)	2,708 (65.6)	
Missing	4,352 (5.2%)			
Parental education level				
One or both parents have higher education	63,492	20,936 (33.0)	42,556 (67.0)	0.01
None of the parents have higher education	8,952	3,070 (34.3)	5,882 (65.7)	
Missing	10,853 (13.0%)			
Family affluence				
Above median score (> 12)	32,537	10,682 (32.8)	21,855 (67.2)	0.73
At or below median score (≤ 11)	30,242	14,701 (32.7)	30,242 (67.3)	
Missing	5,817 (7.0%)			

Statistical tests are Chi Square tests of independence.

### Prevalence of any sexual harassment exposure

The prevalence of having been exposed to any sexual harassment during the past 12 months was 32.6%. As shown in Table 1, the proportions exposed to sexual harassment were significantly higher among girls (39.1%) than boys (25.2%, *p* < 0.001) and also higher for adolescents among whom none of the parents had higher education (34.3%), compared with those where one or both parents had higher education (33.0%, *p* = 0.01). While the test showed a statistically significant association between centrality and sexual harassment exposure, the differences did not conform to a linear pattern. A post-hoc analysis using a dichotomized centrality variable (differentiating the three most central levels from the three least central levels) revealed no significant association with sexual harassment exposure. Thus, centrality was removed from further analysis.

### Exposure to different forms of sexual harassment

The majority of the adolescents reported to not have been exposed to any form of sexual harassment during the past 12 months (between 72 and 86% for the different forms of harassment). For all three harassment types, the proportions of exposed adolescents decreased by each category increase in exposure. Verbal sexual harassment was most frequently reported, with 7.5% of the adolescents reporting six times or more during the past year. The adolescents’ exposure to any sexual harassment, and to each of the sexual harassment forms, is shown in Table 2.

**Table 2:** Adolescents’ exposure to sexual harassment during the last 12 months.

	Number of times exposed			
	1	2–5	≥ 6	Missing
Touching/groping	4,241 (5.1)	2,422 (2.9)	1,377 (1.7)	3,663 (4.4)
Verbal (sexual name-calling)	7,122 (8.6)	6,637 (8.0)	6,214 (7.5)	3,722 (4.5)
Spreading sexual rumors	6,903 (8.3)	3,650 (4.4)	2,124 (2.5)	3,913 (4.7)
	Not exposed (0)	Exposed (≥1)		Missing
Touching/groping	71,594 (86.0)	8,040 (9.7)		3,663 (4.4)
Verbal (sexual name-calling)	59,602 (71.6)	19,973 (24.0)		3,722 (4.5)
Spreading sexual rumors	66,707 (80.1)	12,677 (15.2)		3,913 (4.7)
Any sexual harassment	53,264 (63.9)	25,740 (30.9)		4,293 (5.2)

Table content is number of participants within categories (%). Any sexual harassment is having experienced one or more of the specific harassment forms (touching/groping, verbal or spreading sexual rumors) at least once during the past 12 months.

### Sociodemographic risk factors for sexual harassment exposure

Based on the initial results, gender and parental education were included in the logistic regression analysis as independent predictors of sexual harassment exposure. The model was statistically significant (Model χ^2^ = 1,573, *p* < 0.001, Nagelkerke *r*^2^ = 0.031). The Hosmer-Lemeshow test was not statistically significant, indicating that the model reflected real-world data well. Girls had 90% higher odds of being exposed to sexual harassment during the last 12 months, compared to boys (OR: 1.90, 95% CI: 1.84–1.96). Adolescents among whom at least one parent had higher education had lower odds of sexual harassment exposure, compared to those where none of the parents had higher education (OR: 0.95, 95% CI: 0.90–0.99).

### Psychosocial outcomes associated with sexual harassment exposure

Multivariate effects on the psychosocial outcome variables (depression, loneliness, self-esteem, and subjective well-being) were found for all included independent variables: gender (*p* < 0.001, partial *η*^2^ = 0.11), exposure to sexual harassment (*p* < 0.001, partial *η*^2^ = 0.12), and the gender × sexual harassment interaction term (*p* < 0.001, partial *η^2^* = 0.00).

Proceeding with univariate analyses of predictors of each of the psychosocial outcomes, we found that having been exposed to sexual harassment during the preceding 12 months was significantly associated with all outcomes (partial *η^2^* ranging 0.06–0.11). Adolescents who had experienced sexual harassment had higher levels of depressive symptoms and loneliness, and lower levels of self-esteem and subjective well-being, compared to their counterparts. Similarly, there was a uniform effect of gender. Compared to boys, girls had higher levels of depressive symptoms and loneliness, and lower levels of self-esteem and well-being. The effects of each of the independent variables on each of the psychosocial outcome variables are shown in Table 3.

**Table 3:** Modeled effects of any sexual harassment exposure on psychosocial outcomes.

Independent variables	Depressive symptoms			
	*M*	(95% CI)	*p*	Partial η^2^
Exposure			<0.001	0.11
Exposed	14.66	14.60–14.71		
Not exposed	11.36	11.32–11.39		
Gender			<0.001	0.09
Boys	11.56	11.51–11.61		
Girls	14.46	14.41–14.50		
Exposure × gender			<0.001	0.00
Boys exposed	13.05	12.96–13.14		
Boys not exposed	10.07	10.01–10.12		
Girls exposed	16.26	16.19–16.33		
Girls not exposed	12.65	12.59–12.70		
**Adjusted *r***^**2**^ **= 0.22**				

	Loneliness			
	*M*	(95% CI)	*p*	Partial η^2^
Exposure			<0.001	0.07
Exposed	2.26	2.25–2.27		
Not exposed	1.70	1.69–1.70		
Gender			<0.001	0.04
Boys	1.78	1.77–1.79		
Girls	2.18	2.17–2.19		
Exposure × gender			<0.05	0.00
Boys exposed	2.05	2.03–2.07		
Boys not exposed	1.50	1.49–1.51		
Girls exposed	2.47	2.46–2.49		
Girls not exposed	1.89	1.88–1.90		
**Adjusted *r***^**2**^ **= 0.12**				

	Self-esteem			
	*M*	(95% CI)	*p*	Partial η^2^
Exposure			<0.001	0.06
Exposed	2.77	2.76–2.78		
Not exposed	3.25	3.25–3.26		
Gender			<0.001	0.08
Boys	3.29	3.28–3.30		
Girls	2.74	2.73–2.75		
Exposure × gender			<0.001	0.00
Boys exposed	3.08	3.06–3.09		
Boys not exposed	3.50	3.49–3.51		
Girls exposed	2.47	2.45–2.48		
Girls not exposed	3.01	3.00–3.02		
**Adjusted *r***^**2**^ **= 0.15**				

	Well-being			
	*M*	(95% CI)	*p*	Partial η^2^
Exposure			<0.001	0.06
Exposed	6.53	6.51–6.55		
Not exposed	7.52	7.50–7.53		
Gender			<0.001	0.04
Boys	7.44	7.42–7.46		
Girls	6.61	6.59–6.63		
Exposure × gender			<0.001	0.00
Boys exposed	7.00	6.97–7.04		
Boys not exposed	7.88	7.85–7.90		
Girls exposed	6.06	6.03–6.09		
Girls not exposed	7.16	7.14–7.19		
**Adjusted *r***^**2**^ **= 0.11**				

Exposure is any exposure to one or more forms of sexual harassment during the last 12 months.

**Figure 1:**
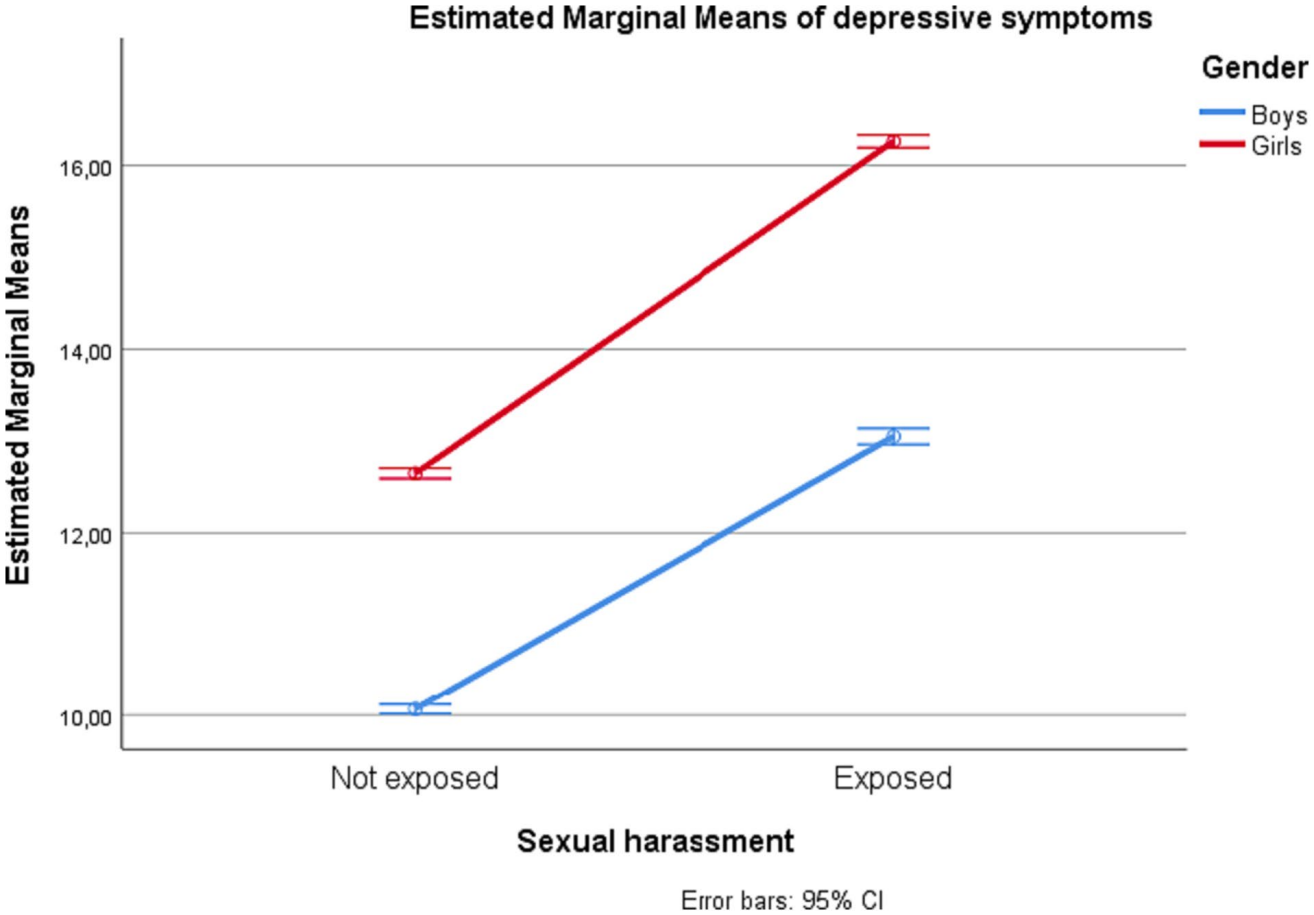
Depressive symptoms among adolescents in junior high school with and without exposure to sexual harassment by gender.

**Figure 2:**
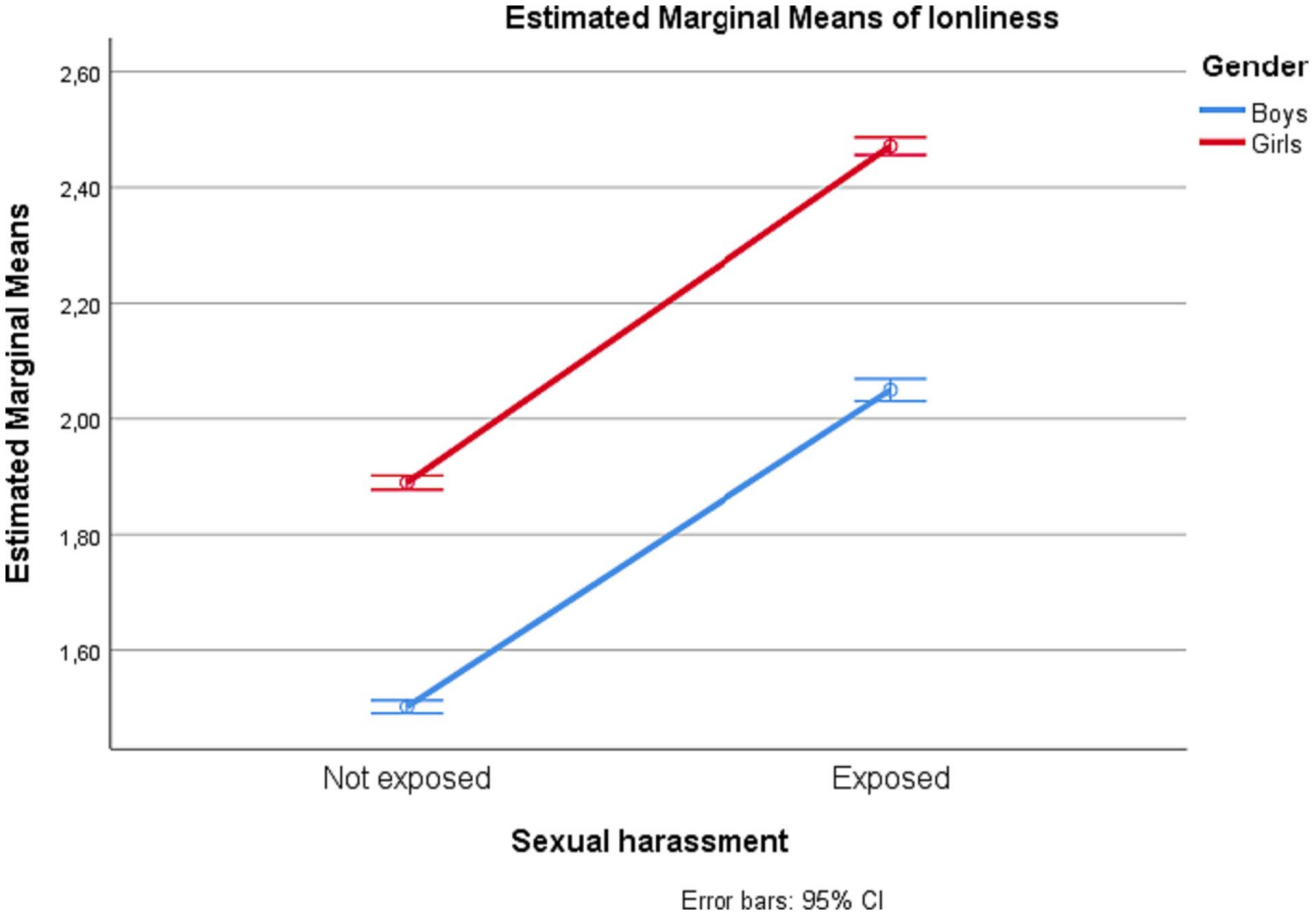
Loneliness among adolescents in junior high school with and without exposure to sexual harassment by gender.

**Figure 3:**
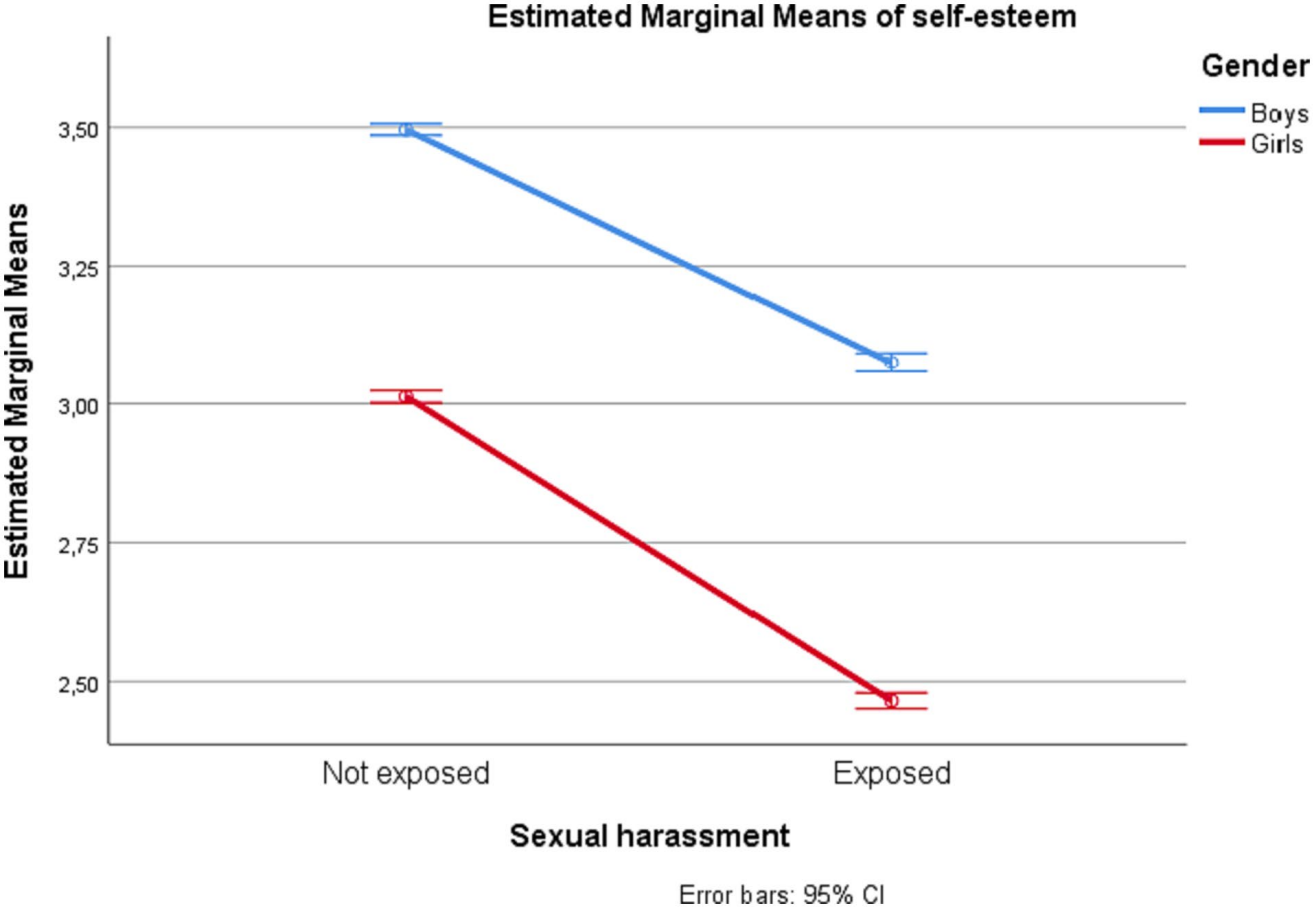
Self-esteem among adolescents in junior high school with and without exposure to sexual harassment by gender.

**Figure 4:**
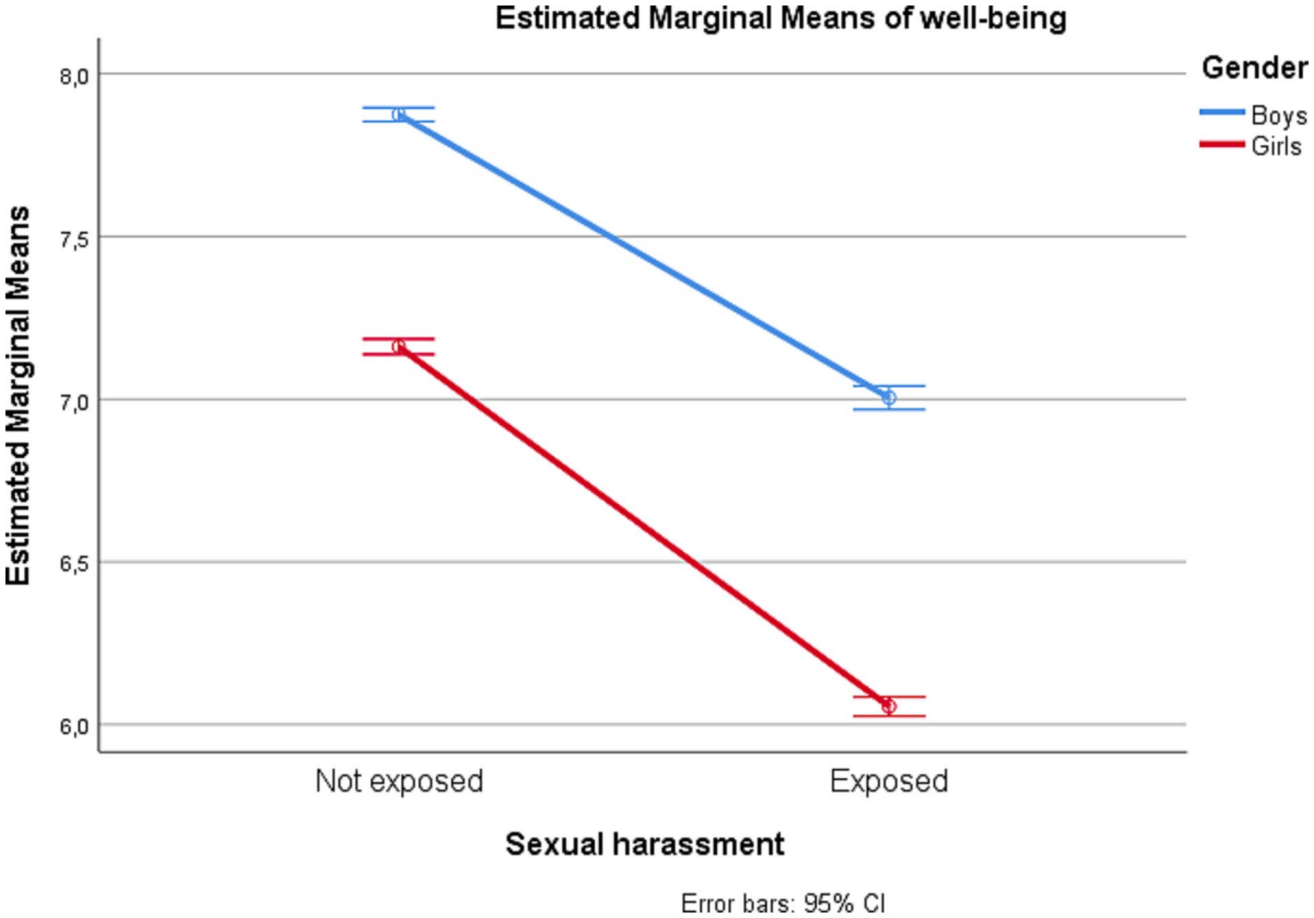
Subjective well-being among adolescents in junior high school with and without exposure to sexual harassment by gender.

Gender interacted significantly with sexual harassment exposure for all outcome estimates, but with negligible effect sizes (partial *η^2^* = 0.00). The estimated marginal means for depressive symptoms, loneliness, self-esteem, and subjective well-being among adolescents with and without exposure to sexual harassment are displayed by gender in Figures 1–4.

### Relationships between frequency of exposure and psychosocial factors

The results from the linear regression analyses showing associations between frequency of sexual harassment exposure and levels of depressive symptoms, loneliness, self-esteem, and well-being, are displayed in the Supplementary Table S1. More frequent exposure to all three forms of harassment was associated with higher levels of depressive symptoms, with β ranging between 0.13–0.22 for the three forms (all *p* < 0.001). More frequent exposure to touching/groping and to verbal harassment were slightly more strongly associated with higher levels of depressive symptoms for girls (β = 0.16 and 0.22 for touching/groping and verbal harassment, respectively; both *p* < 0.001) than for boys (β = 0.11 and 0.17, respectively; both *p* < 0.001). Despite a statistically significant interaction between exposure to sexual rumors and gender, the effect sizes were identical for both genders (β = 0.21, *p* < 0.001).

More frequent exposure to all three forms of harassment was also associated with higher levels of loneliness (β ranging between 0.13–0.22, all *p* < 0.001). All associations interacted significantly by gender, although effect sizes for the association between touching/groping and loneliness were identical for boys and girls (β = 0.10, *p* < 0.001). For verbal harassment, the association with loneliness was slightly stronger for girls than for boys (β = 0.16 versus β = 0.12, both *p* < 0.001). For sexual rumors, the association was practically identical for boys and girls (β = 0.19 versus β = 0.17, both *p* < 0.001).

More frequent exposure to all harassment forms was associated with lower levels of self-esteem, with β ranging between −0.09 and − 0.15 (all *p* < 0.001). All interaction terms were statistically significant (*p* < 0.001). Subsequent gender-specific analyses revealed that the association between touching/groping and self-esteem was slightly stronger for girls than for boys (β = −0.10 versus β = −0.06, both *p* < 0.001), as was the association between verbal harassment and self-esteem (β = −0.17 versus β = −0.12, both *p* < 0.001). The effect sizes for the association between sexual rumors and self-esteem was practically identical between girls and boys (β = −0.13 versus β = −0.14, respectively, both *p* < 0.001).

Similarly, more frequent exposure to all forms of harassment was associated with lower levels of well-being, with β ranging between −0.14 and − 0.20 (all *p* < 0.001). All interaction terms were statistically significant (*p* < 0.001). Gender-specific analyses showed that the association between touching/groping and well-being was fairly similar for girls and boys (β = −0.13 versus β = −0.10, both *p* < 0.001), while the association between verbal harassment and well-being was slightly stronger for girls (β = −0.19 versus β = −0.13, both *p* < 0.001). The effect sizes for the association between sexual rumors and well-being was identical between girls and boys (β = −0.17, *p* < 0.001).

Considering the one-item scales with restricted scale range used for measuring loneliness and self-esteem, we also tested a series of ordinal regression models attempting to verify the dose–response relationships with more robust methods. The results are displayed in the Supplementary Table S2. For all three types of sexual harassment, more frequent exposure was consistently associated with higher levels of loneliness and lower levels of self-esteem. Thus, dose–response relationships between sexual harassment exposure and these outcomes were established also with this analysis.

## Discussion

### Main summary of results

While the majority of adolescents reported not to have been exposed to any form of sexual harassment, 32.6% had experienced at least one form of sexual harassment during the past 12 months. Girls had almost twice as high risk of having been exposed to sexual harassment compared to boys, while adolescents who had at least one parent with higher education had a lower chance of having been exposed. Adolescents who had been exposed to sexual harassment had higher levels of depressive symptoms and loneliness, and lower levels of self-esteem and subjective well-being, compared to non-exposed adolescents. Among adolescents who had been exposed to some form of sexual harassment, higher frequency of exposure was associated with poorer psychosocial outcomes in all areas.

### Sexual harassment prevalence and risk factors

In this study of junior high-school students, one out of three had experienced sexual harassment during the past year. Previous studies of adolescents have used diverse samples and a variety of measurement methods and also reported varying prevalence rates. For example, studies from USA have found prevalence rates of 25.0% (11) and 36.3% (45), whereas a study from Scotland reported a prevalence of 64.7% for any sexual harassment (12). A study from Norway also reported prevalence rates exceeding 60% for both boys and girls in 2014 (5), whereas a Norwegian study using data from 2018 showed lower rates for both boys (26.5%) and girls (36.1%) (15). Considering these studies there may be a time trend of reduced prevalence of sexual harassment, possibly due to public awareness campaigns such as the #MeToo movement. However, the prevalence rates of our study are within the range of findings reported in many national and international studies. Jointly the findings imply that sexual harassment affects a substantial proportion of young adolescents in junior high school. In line with the international literature (6, 12–14), girls are exposed to a greater extent than boys, as demonstrated by the almost twice as high odds among girls for having been exposed to sexual harassment.

We found no association between family affluence and sexual harassment exposure but higher education among one or both parents was associated with lower odds of exposure to sexual harassment. Possibly, higher education experience among parents may translate into more protective attitudes and behavior in relation to their adolescent, such as suggesting or otherwise influencing activities, friends and leisure time arenas that may constitute a lower risk of harassment exposure. Similar findings have been demonstrated in a previous study, suggesting that parental involvement in adolescents’ lives may be protective against sexual harassment (22). Moreover, a longitudinal cohort study found that higher parental education levels predicted better mental health among their children at two-year follow-up, and that stressful life situations were more frequent in families with low socioeconomic status (46). These findings may indicate that higher parental education levels serve as an indirect barrier, via more protective parental behaviors, for adolescents’ exposure to problematic situations such as sexual harassment. In turn, lower chance of sexual harassment exposure may protect adolescents’ mental health.

### Psychosocial factors associated with sexual harassment exposure

The results showed that adolescents exposed to sexual harassment had consistently poorer scores across all psychosocial outcomes compared to their non-exposed counterparts. Moreover, girls had consistently poorer scores across all psychosocial outcomes compared to boys. Several studies have reported associations between sexual harassment exposure and mental health problems, such as depressive symptoms (6, 13–15, 47), anxiety symptoms (14, 47, 48), alcohol and drug use (6, 49) and emotional problems more in general (18). Using a cross-lagged model, Skoog and Kapetanovic (18) also found that sexual harassment exposure and emotional problems were related in a transactional manner – sexual harassment at baseline predicted emotional problems at the first follow-up, whereas emotional problems at the first follow-up predicted sexual harassment exposure at the second follow-up. These findings suggest that mental health problems can be both cause and effect of sexual harassment experience. In line with results from our study, these researchers (18), also confirmed that gender moderated the associations between sexual harassment exposure and the psychosocial outcomes, as exposure to sexual harassment had a stronger negative impact on girls than boys. However, the results from our study indicate that the moderating effects of gender were very small.

### Relationship between frequency of exposure and psychosocial outcomes

Finally, we found a dose–response relationship between higher frequency of sexual harassment exposure and poorer psychosocial outcomes in all areas. Similarly, Heir and co-workers’ study of the general Norwegian population showed that having experienced a greater number of traumatic events was related to higher likelihood of symptom-defined post-traumatic stress disorder (50). Moreover, the results reported by Collinsworth et al. (29) demonstrated higher frequency of harassment to be related to higher prevalence of psychological distress. Thus, these studies support the results of our study in indicating dose–response relationships – although the effect sizes were relatively small – between frequency of negative experiences and psychosocial outcomes. Moreover, girls reported higher frequency of sexual harassment exposure than boys, and thus experienced stronger negative effects, but again, effect sizes were small.

### Study strengths and limitations

The high participation rate (44), the large sample size, and the use of recently collected data (2021) are major strengths of the present study. However, the very large sample size, resulting in high statistical power, requires that the effect sizes of statistically significant results are considered. In this study, statistically significant interaction effects of sexual harassment exposure and gender on psychosocial outcomes were generally small.

A limitation of this study is the cross-sectional design, which precludes inferences about causal relationships. Depressive symptoms, feelings of loneliness, low self-esteem and poor well-being may not only be a result of exposure to sexual harassment; such psychosocial problems may also increase the risk of experiencing sexual harassment. Indeed, a self-strengthening “intertwined” development of sexual harassment exposure and emotional problems was found in a cross-lagged longitudinal study (18).

To an extent, the study relies on self-report measures, some of which with unknown psychometric properties. Thus, the validity of these measures may be questioned. In addition, we classified participants with “any sexual harassment” if they had been exposed to either sexual name-calling, sexual rumors, touching/groping, or several of these. However, sexual harassment may include a range of different behaviors that were not included in these items. Therefore, there is a risk that the actual prevalence of sexual harassment was underestimated in this study. While the questions used to assess sexual harassment have been used in the Ungdata surveys for years (32), they have also changed over time. It is fully possible that adolescents’ views on sexual harassment would suggest a further development of the questions that are asked to address this issue. As indicated from a previous study, focus groups might be a good method by which to elicit relevant information based on adolescents’ own views (51). Furthermore, the associations found between sexual harassment and the psychosocial outcomes may be influenced by other factors not controlled for in the study. The perceived severity of sexual harassment incidents has been found to be the dominating predictor of psychological distress (29), but unfortunately, severity was not assessed in our study. Similarly, the study did not assess the relationship between victims and perpetrators of sexual harassment, nor was the location of incidents of sexual harassment assessed.

## Conclusion and implications

Among adolescents in junior high school, about one-third had been exposed to some form of sexual harassment during the past year, and girls were twice as often exposed compared to boys. Overall, sexual harassment exposure was associated with poorer psychosocial outcomes, including higher levels of depression and loneliness and lower levels of self-esteem and well-being. In addition, among those exposed, we found a dose–response relationship between higher frequency of exposure and poorer psychosocial outcomes.

Sexual harassment is pervasive in society, and given the negative health effects on those exposed, it constitutes a serious public health problem. Establishing and sustaining safe and healthy environments for children and adolescents on the arenas where they live, play, study, and socialize, is therefore of great importance. An increased awareness of sexual harassment and its consequences among same-aged peers and in the general public may assist in reaching this aim. However, a particular responsibility for prevention and intervention lies on adults and key personnel interacting with children and adolescents on a day to day basis, such as parents, teachers, health personnel, social workers, and leaders of organized leisure activities. Responsible adults in these key groups should promote a culture of respect that may prevent sexual harassment to occur. This may be done by setting explicit norms of conduct and ways of speaking to each other, and not least by openly discussing the reasons for upholding such norms while accepting the diversity of responses to them. In line with self-determination theory (52), conduct norms have been found to be more easily accepted when the reasons for them are clear and when the norms are conveyed with an autonomy-supportive stance, leading to a sense of shared ownership to them (53). However, when adults do gain knowledge of instances of sexual harassment, they should make sure that the exposed person receives adequate support and that the problem is addressed. The study also suggests that adolescents who are repeatedly exposed to sexual harassment should receive special attention and support, as they are likely to suffer the worst psychosocial outcomes in terms of depressive symptoms, loneliness, reduced self-esteem and lower well-being.

Digital communication forms and the use of various social media are gaining ground, potentially creating new arenas for bullying and harassment among adolescents. Future studies should also explore the extent to which the digital social arenas also become arenas for sexual harassment, and how digital harassment may interact with traditional forms. Moreover, future research should make sure to elicit adolescents’ conceptualizations of sexual harassment, and to incorporate this knowledge in assessment instruments and interview schedules. Prevention initiatives to sustain safe social environments for adolescents should be carefully evaluated, as should individualized interventions for adolescents who are victims or perpetrators of sexual harassment.

## Data availability statement

The data analyzed in this study is subject to the following licenses/restrictions. The data and materials from the Ungdata surveys are stored in a national database administered by NOVA. The data are available for research purposes upon application. For request of the data, please contact ungdata@oslomet.no. Further information about the study and the questionnaires can be found at: https://www.nsd.no/nsddata/serier/ungdata_eng.html. Requests to access these datasets should be directed to ungdata@oslomet.no.

## Ethics statement

The studies involving humans were approved by Research Ethics Committee at Inland Norway University of Applied Sciences. The studies were conducted in accordance with the local legislation and institutional requirements. Written informed consent for participation was not required from the participants or the participants’ legal guardians/next of kin because before participating in the study, all adolescents were asked to provide informed consent. The parents received oral and written information about the study and were given the opportunity to withdraw their children from participation. The information letter was approved by The Norwegian Centre for Research Data (NSD).

## Author contributions

TB: Conceptualization, Data curation, Formal analysis, Funding acquisition, Investigation, Methodology, Project administration, Validation, Visualization, Writing – original draft, Writing – review & editing. AS: Conceptualization, Investigation, Methodology, Writing – review & editing. MG: Conceptualization, Investigation, Methodology, Writing – review & editing. CD: Conceptualization, Investigation, Methodology, Writing – review & editing. TS: Conceptualization, Investigation, Methodology, Writing – review & editing.
