# Supplementary material for: Sexual harassment exposure among junior high school students in Norway: prevalence and associated factors
Source: Front Public Health. 2024 Jan 18;12:1307605. doi: 10.3389/fpubh.2024.1307605 (PMC10830835; doi:10.3389/fpubh.2024.1307605)
Supplement: Supplementary file 1 [file Data_Sheet_1.PDF]

Supplementary file

Table 1. Linear regression analysis displaying associations between frequency of exposure and psychosocial factors among adolescents exposed to sexual harassment

	Depressive symptoms			
Independent variables	*β*	*p*	*β*	*p*
Model 1				
Touching/groping	0.13	<0.001	-0.11	<0.001
Touching/groping × gender			0.33	<0.001
Model 2				
Verbal harassment	0.16	<0.001	-0.10	<0.001
Verbal harassment × gender			0.42	<0.001
Model 3				
Rumor spreading	0.22	<0.001	-0.06	<0.001
Rumor spreading × gender			0.39	<0.001
	Loneliness			
Independent variables	*β*	*p*	*β*	*p*
Model 1				
Touching/groping	0.11	<0.001	-0.02	0.07
Touching/groping × gender			0.16	<0.001
Model 2				
Verbal harassment	0.13	<0.001	-0.01	<0.001
Verbal harassment × gender			0.22	<0.001
Model 3				
Rumor spreading	0.19	<0.001	0.04	<0.001
Rumor spreading × gender			0.20	<0.001
	Self-esteem			
Independent variables	*β*	*p*	*β*	*p*
Model 1				
Touching/groping	-0.09	<0.001	0.12	<0.001
Touching/groping × gender			-0.27	<0.001
Model 2				
Verbal harassment	-0.12	<0.001	0.09	<0.001
Verbal harassment × gender			-0.33	<0.001

Model 3				
Rumor spreading	-0.15	<0.001	0.09	<0.001
Rumor spreading × gender			-0.33	<0.001
		Well-being		
Independent variables	*β*	*p*	*β*	*p*
Model 1				
Touching/groping	-0.14	<0.001	0.01	0.21
Touching/groping × gender			-0.18	<0.001
Model 2				
Verbal harassment	-0.15	<0.001	0.02	<0.05
Verbal harassment × gender			-0.26	<0.001
Model 3				
Rumor spreading	-0.20	<0.001	-0.02	0.08
Rumor spreading × gender			-0.23	<0.001

Note. Gender codes: 1 = boys, 2 = girls.

Supplementary file

Table 2. Ordinal regression analyses displaying associations between frequency of exposure and loneliness and self-esteem among adolescents exposed to sexual harassment

		Loneliness	
Independent variables	Estimate	95% CI	*p*
Touching/groping			
Once	-0.52	-0.63- -0.41	<0.001
2-5 times	-0.25	-0.37- -0.13	<0.001
6 or more times		reference	
Verbal harassment			
Once	-0.64	-0.71- -0.58	<0.001
2-5 times	-0.27	-0.34- -0.21	<0.001
6 or more times		reference	
Rumor spreading			
Once	-0.96	-1.05- -0.87	<0.001
2-5 times	-0.55	-0.65- -0.45	<0.001
6 or more times		reference	
		Self-esteem	
Independent variables	Estimate	95% CI	*p*
Touching/groping			
Once	0.35	0.24-0.46	<0.001
2-5 times	0.17	0.05-0.29	<0.01
6 or more times		reference	
Verbal harassment			
Once	0.57	0.51-0.63	<0.001
2-5 times	0.17	0.10-0.23	<0.001
6 or more times		reference	
Rumor spreading			
Once	0.69	0.60-0.78	<0.001
2-5 times	0.33	0.24-0.43	<0.001
6 or more times		reference	

Note. Sexual harassment frequency indicates how many times during the past 12 months.
